# Endothelin B Receptors on Primary Chicken Müller Cells and the Human MIO-M1 Müller Cell Line Activate ERK Signaling via Transactivation of Epidermal Growth Factor Receptors

**DOI:** 10.1371/journal.pone.0167778

**Published:** 2016-12-08

**Authors:** Mohammad Harun-Or-Rashid, Dardan Konjusha, Caridad Galindo-Romero, Finn Hallböök

**Affiliations:** Department of Neuroscience, Uppsala University, Uppsala, Sweden; University of Michigan, UNITED STATES

## Abstract

Injury to the eye or retina triggers Müller cells, the major glia cell of the retina, to dedifferentiate and proliferate. In some species they attain retinal progenitor properties and have the capacity to generate new neurons. The epidermal growth factor receptor (EGFR) system and extracellular signal-regulated kinase (ERK) signaling are key regulators of these processes in Müller cells. The extracellular signals that modulate and control these processes are not fully understood. In this work we studied whether endothelin receptor signaling can activate EGFR and ERK signaling in Müller cells. Endothelin expression is robustly upregulated at retinal injury and endothelin receptors have been shown to transactivate EGFRs in other cell types. We analyzed the endothelin signaling system in chicken retina and cultured primary chicken Müller cells as well as the human Müller cell line MIO-M1. The Müller cells were stimulated with receptor agonists and treated with specific blockers to key enzymes in the signaling pathway or with siRNAs. We focused on endothelin receptor mediated transactivation of EGFRs by using western blot analysis, quantitative reverse transcriptase PCR and immunocytochemistry. The results showed that chicken Müller cells and the human Müller cell line MIO-M1 express endothelin receptor B. Stimulation by the endothelin receptor B agonist IRL1620 triggered phosphorylation of ERK1/2 and autophosphorylation of (Y1173) EGFR. The effects could be blocked by Src-kinase inhibitors (PP1, PP2), EGFR-inhibitor (AG1478), EGFR-siRNA and by inhibitors to extracellular matrix metalloproteinases (GM6001), consistent with a Src-kinase mediated endothelin receptor response that engage ligand-dependent and ligand-independent EGFR activation. Our data suggest a mechanism for how injury-induced endothelins, produced in the retina, may modulate the Müller cell responses by Src-mediated transactivation of EGFRs. The data give support to a view in which endothelins among several other functions, serve as an injury-signal that regulate the gliotic response of Müller cells.

## Introduction

**Fig 1:**
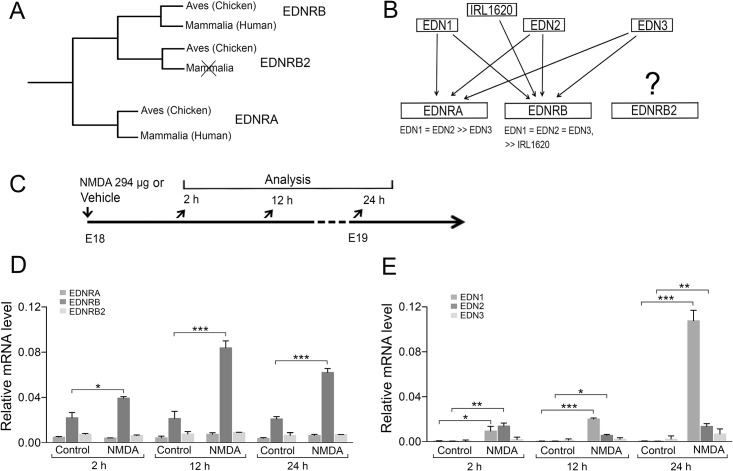
Endothelins and their receptors in retina after excitotoxic injury. (A) Schematic tree depicting orthologs and paralogs of the endothelin receptors (EDNRs) in Aves and Mammalia. EDNR2B has only been found in non-mammalian species. The tree is based on Ensembl Gene tree ID: ENSGT00760000119177. (B) Interactions between the endothelins (EDNs), the EDNRB agonist IRL1620 and the EDNRs. (C) Experimental outline. QRT-PCR analysis of (D) EDNRA, EDNRB, EDNRB2 and (E) EDN1, EDN2 and EDN3 mRNA levels in NMDA- or vehicle- (Control) treated eyes. Bar graphs show the relative mRNA levels normalized to ß-actin. Bar graphs are mean ± SEM, n = 6 (control 2 h), n = 5 (NMDA 2 h), n = 6 (control 12 h), n = 5 (NMDA 12 h), n = 6 (control 24 h), n = 6 (NMDA 24 h, (*P < 0.01, **P < 0.001, ***P< 0.0001) analyzed by one-way ANOVA and Tukey’s post hoc test. Significance is only indicated for the comparisons: control-NMDA at 2h, 12h and 24h.

Glia cells control homeostasis and support neuronal survival after neural injury but they may also serve as progenitor cells and in some systems contribute to retinal regeneration. The endogenous regulation of the glia cell response after injury is therefore important for the outcome after injury. In this work we have studied the intracellular signal transduction response in retinal Müller glia with focus on mitogen activated protein kinase (MAPK)/extracellular signal-activated kinases 1/2 (ERK1/2)-signaling, triggered by endothelins (EDNs). EDNs are best known for their potent vasoconstrictive activity but they have direct effects on both neurons and glia cells in the developing and adult nervous system [1–3]. The EDNs are encoded by three genes: *EDN1*, *EDN2* and *EDN3*. The active peptides are generated as prepro-endothelin peptides that are proteolytically processed to 21 amino acid mature endothelins. EDNs have distinct binding properties to two main receptors; endothelin receptor A (EDNRA) and endothelin receptor B (EDNRB) [4, 5]. A third endothelin receptor (EDNRB2) has been found only in non-mammalian vertebrates but it is less well characterized than EDN1 and EDN2 (Fig 1A and 1B) [6]. The EDNRs are seven transmembrane domain G-protein-coupled receptors (GPCRs) that activate different signaling systems depending on what cell type the receptor is expressed in. They couple to members of the Gi, Gq, Gs, and Gα12/13 G-protein families [7] and activation leads to modulation of several effectors including adenyl cyclase, phospholipase C, cyclooxygenases, nitric oxide synthase, phosphatidylinositide 3-kinase and in some cells they also trigger ERK1/2 signaling [8–10].

Cells in the retina predominantly express EDN1 and EDNRB. They are expressed in photoreceptors, inner nuclear layer cells including Müller cells and cells in the ganglion cell layer [3]. Different retinal injuries upregulate both EDNRA and EDNRB, as well as EDN1 and EDN2 [3], and a growing body of data suggests roles in retinal pathogenesis including diabetic retinopathy and glaucoma [11]. EDN1 is elevated in aqueous humor of some glaucoma patients [12–14] and EDN1 has been shown to cause retinal ganglion cell death in experimental models for glaucoma [15, 16]. Opposed to the adverse effects seen by EDN1 in several injury models, EDN2 has displayed neuroprotective properties for photoreceptors. Over-expression of EDN2 in a mouse model for photoreceptor degeneration rescued photoreceptors [17]. The EDNRB antagonist BQ-788 increase inherited photoreceptor loss, while the agonist, BQ-3020, reduced photoreceptor loss after light-induced injury [18]. Over-expression of Norrin in the retinal pigment epithelium, which protects photoreceptors is associated with up-regulation of EDN2 expression in retina [19]. Phototoxic injury upregulates EDN2 in photoreceptors and EDNRB in the Müller cells [3] and EDN2 has therefore been suggested to mediate signaling between degenerating photoreceptors and Müller cells [3].

Müller cells maintain and protect retinal neurons [20], and they contribute to retinal regeneration in many non-mammals by dedifferentiating to retinal progenitor cells and subsequent formation of new retinal neurons. This process is dependent on the activation of ERK signaling downstream of EGF receptors (EGFRs) [21]. EDNRBs have been shown to transactivate EGFRs in vascular smooth muscle cells [7, 22]. The EGFR transactivation requires activation of Src-kinase and matrix metalloproteinases (MMPs). Transactivation engages the release of the heparin binding-EGF (HB-EGF) that stimulates EGFR on the same cells in an autocrine mode of action. Müller cells express both EGFR and HB-EGF [21, 23], but it is not known whether stimulation of EDNRB transactivates the EGFR signaling in Müller cells.

In this work, we tested the hypothesis that stimulation of EDNRB by an EDNRB agonist elicits transactivation of EGFRs and ERK1/2 signaling in Müller cells. We studied this in both chicken Müller cells and in a human Müller cell-line; MIO-M1 [24]. First, we studied the expression of the endothelins and their receptors in chicken retina and confirmed their response to injury in the system. We used excitotoxic injury of the late embryonic chicken retina that is known to robustly activate Müller cells. EDNRs were expressed in both chicken primary Müller cells and in the MIO-M1 cells and stimulation of EDNRs on Müller cells using the EDNRB agonist IRL1620 [25] induced a robust ERK response. The ERK response was used to monitor EDNR-triggered Src- and MMP-dependent activation of EGFR in Müller cells. Our results showed that both chicken and human Müller cells expressed EDNRB and that stimulation by IRL1620 caused both Src-kinase mediated ligand-dependent and ligand-independent EGFR signaling that is indicatory for EGFR transactivation. These results implicate that injury-induced EDN-signaling modulate the Müller cell response that include transactivation of EGFRs.

## Materials and Methods

### Animals

Fertilized White Leghorn (local breed) chicken eggs were obtained from OVA Produktion AB (Västerås, Sweden) and incubated at 38°C in a humidified egg-incubator (Grumbach, Asslar, Germany). All animal experiments were performed according to the recommendations and the guidelines given by ARVO statements for the use of animals in ophthalmic and vision research and was approved by the local ethics committee in Uppsala (Uppsala djurförsöksetiska nämnd).

### Intra-ocular injection

Intra-ocular injections were made on embryonic day (E) 18 embryos in the dorsal quadrant of the eye using a Hamilton syringe (Bonaduz, Switzerland) with 27-G needle. A small hole was made in the eggshell and chorioallantoic membranes, head was pulled up with a bent glass rod and injections were made through the membranes. Ten microliter (10 μg) of IRL1620 or 20 μl (294 μg) of N-methyl-D-aspartate (NMDA) in sterile saline solution (0.15 M NaCl) was injected into the experimental right eye (the reagents are listed in S1 Table). For control experiment, saline solution (vehicle) was injected. After the injections, eggs were sealed and incubated for different periods of times as indicated in the figures and then analyzed.

### Müller cell cultures

Primary chicken Müller cell cultures were established as previously described [23]. Briefly, 12 E14 chick eyes were enucleated and retinas were dissected, dissociated and cultured in Dulbecco’s modified Eagle’s medium (DMEM) with 10% (Newborn calf serum (NCS), 2 mM glutamine, 100 U/mL penicillin, and 100 mg/mL streptomycin at 37°C. Cultures were fed three times in a week up to 4 weeks. Primary cultures were ready to use when all neurons were gone and the cultures only contained Müller cells. The cell purity was determined by immunostaining with chicken Müller cell-specific antibody, 2M6 and purity was found more than 95%. Human Müller cell-line, Moorfields/Institute of Ophthalmology-Müller 1 (MIO M1) was obtained through University College London Business’s (UCLB) on-line licensing system (E-LUCID, London, UK). The MIO-M1 cell-line was cultured in DMEM with 10% NCS, 2 mM glutamine, 100 U/mL penicillin, and 100 mg/mL streptomycin at 37°C. Media were changed twice in a week and cells were ready to use when reached to more than 90% confluency. Prior to cell cultures treatments, chick primary Müller cells and human Müller cell line were serum-starved for 5 and 16 h respectively. Serum-starved Müller cells were supplemented with IRL1620 (5 μM), EGF (100 ng/mL), or specific inhibitors: BQ-788 (50 μM), AG1478 (50 μM), GM6001 (50 μM), PP1 (5 μM), and PP2 (5 μM) (S1 Table). For control experiments, cells were treated with vehicles.

### Cell transfection with small interfering RNA (siRNA)

We used siRNA to knock-down the expression of EGFR and human MIO-M1 cells plated in 6-well dishes were transfected in the absence of serum and antibiotics with non-specific target (Stealth RNAi Negative Control Duplex, Cat # 12935–300, Invitrogen, Carlsbad, CA) or EGFR-siRNA (5′-GGAUCCCAGAAGAAGGUGAGAAAGUUAA- 3′, Accession no. NM_005228.3) with Lipofectamine RNAi-MAX (Cat # 13778–030, Invitrogen, Carlsbad, CA) according to the manufacturer’s instructions. After 48 h of EGFR siRNA transfection, cells were treated with IRL1620 (5 μM) or vehicle and were analyzed after 10 min for effects on EGFR, phospho-EGFR (Y1173) and phospho-ERK1/2 levels.

### Immunohistochemistry, cytochemistry and microscopy

Enucleated eyes were fixed in 4% paraformaldehyde and frozen in NEG 50^®^ freezing medium (Thermo Scientific, Kalamazoo, MI, USA) and sectioned at10 μm using a cryostat. Immunohistochemistry and cytochemistry were performed as described previously [23, 26]. Details of primary and secondary antibodies are listed in the S2 Table. For microscopy, Zeiss Axioplan2 microscope integrated with Axiovision software v4.8 (CarlZeiss GmbH, Hamburg Germany) was used. Photomicrographs were captured from the central part of the retina and the same setting of exposure time was used while capturing the photomicrographs for both the experimental and control groups.

### Quantitative reverse transcriptase-PCR (qRT-PCR)

Total RNA was isolated with TRIzol (Invitrogen, Carlsbad, CA, USA) and cDNA was synthesized from 1 μg DNase-treated RNA by using High Capacity RNA to cDNA synthesis kit (Applied Biosystems, Foster City, CA, USA). QRT-PCR analysis (IQ SyBr Green Supermix and a C1000 Thermal Cycler; Bio-Rad, Hercules, CA, USA) was performed as previously described [23, 26]. QRT-PCR primers were designed by using Primer Express v2.0 software (Applied Biosystems, Foster City, CA, USA). The mRNA expression levels were normalized to β-actin expression levels. The use of β-actin for normalization purposes has been validated by checking the most stable mRNA expression of glyceraldehyde-3-phosphate dehydrogenase (GAPDH), TATA binding protein (TBP), β-2-microglobulin, and β-actin using geNorm [27]. Primer efficiency, linearity and specificity were checked (S1 and S2 Figs) and the expression levels were calculated from cycle threshold (Ct) and 2^-ΔΔCt^ method [28]. Primers sequences are listed in the S3 Table.

### Western blot and statistical analyses

Retinas were dissected from the enucleated eyes or Müller cells were scraped off the petri dish and homogenized in the lysis buffer containing Halt Protease and Phosphatase Inhibitor Cocktail (Thermo Scientific, Rockford, IL, USA). The total protein concentration was measured by using Dc Protein Assay kit (Bio-rad, Hercules, CA, USA). The Western blot analysis was performed as previously described [23, 29] and followed the manufacturer’s instructions (Bio-rad). For protein densitometry, Image Lab v4.1 software was used (Bio-rad). Details of primary and secondary antibodies are listed in the S2 Table. For statistical analysis, GraphPad Prism 6 (GraphPad Software Inc. La Jolla, CA, USA) software was used and the data were analyzed by one-way ANOVA and Tukey’s multiple comparison post hoc test.

## Results

### Confirmation of injury-induced expression of EDNRB and its ligands EDN1 and EDN2 in E18 chicken retina

The expression of EDNRB and its ligands are induced in different models of photoreceptor disease or injury. Acute light damage causes more than a 10-fold increase of EDNRB expression in mouse Müller cells 24 h after injury [3] and we investigated whether the expression of EDNs and EDNRs was induced after excitotoxic injury. The injury was induced by intra-ocular injection of NMDA in E18 chicken retina. The relative mRNA levels of EDNRs and EDNs were analyzed by using qRT-PCR at 2, 12, and 24 h after an intra-ocular injection of NMDA (Fig 1C). The mRNA levels of EDNRB were significantly increased at 2 h with very high levels at 12 and 24 h after NMDA treatments (Fig 1D). The mRNA expression of EDNRA and EDNRB2 was unaffected at the 12 and 24 h time points after NMDA treatments (Fig 1D). The mRNA expression of the endothelins EDN1 and EDN2 significantly increased at 2, 12, and 24 h after NMDA treatments (Fig 1E). While the EDN3 expression levels were unchanged (Fig 1E). The EDNRB2 gene is not present in mammals (Fig 1A). These results confirm that the expression of EDNRB and its ligands EDN1 and EDN2 is robustly increased after excitotoxic injury to chicken retina.

### Activation of ERK1/2 in E18 chicken retina after EDNRB stimulation

**Fig 2:**
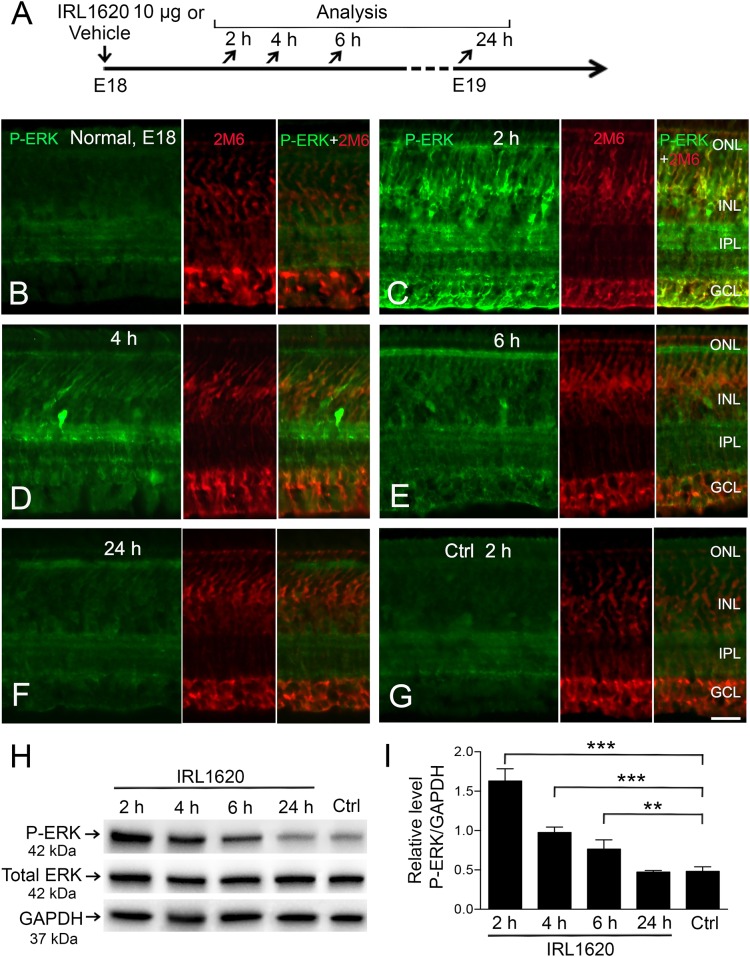
EDNRB agonist IRL1620 activates P-ERK1/2 in chicken retina. Immunohistochemistry and western blot analysis of P-ERK after intra-ocular injection of IRL1620 in E18 chicken embryo. (A) Experimental outline. (B–G) Fluorescence micrographs showing P-ERK and 2M6 (Müller cell marker) immunoreactivity in (B) normal untouched retina, retina after (C) 2 h, (D) 4 h, (E) 6 h, (F) 24 h IRL1620 treatment. (G) Vehicle-injected eye at 2 h (Ctrl). (H) Representative western blot analysis of P-ERK in retina 2, 4, 6, and 24 h after IRL1620 treatment. Note that western blot analysis for ERK1/2 in chicken only shows one band in contrast to the two bands that are seen in mammals (I) Bar graph with densitometry of P-ERK levels normalized by GAPDH levels. Normalization to total ERK gave similar results (S1 Fig). Bar graph is mean ± SEM, n = 3 (**P < 0.001, ***P < 0.0001) analyzed by one-way ANOVA and Tukey’s post hoc test. Significance is only indicated for comparisons from control 2 h to IRL1620 2 h, 4 h and 6 h. Scale bar in (G) is 20 μm, also valid for (B–F).

We determined the dose-response of the EDNRB agonist IRL1620 that gave an increased phosphorylation of ERK1/2 in the chicken retina (S3 Fig). The phosphorylation of ERK1/2 was studied by using immunohistochemistry for phosphorylated-ERK1/2 (P-ERK) in combination with the Müller cell marker, 2M6 [30, 31] (P-ERK, 2M6 double positive cells) at 2, 4, 6, and 24 h after intra-ocular injection of the EDNRB agonist IRL1620 (Fig 2A). The effective dose was 5 μg IRL 1620 (S3 Fig). P-ERK immunoreactivity (IR) was seen in 2M6+ cell-processes in the vitreal end-feet in the nerve fiber layer, in 2M6+ somata in the inner nuclear layer, and in 2M6+ cell-processes in the photoreceptors layer (Fig 2C and 2D). There was no increase of P-ERK IR in control retinas injected with vehicle. The P-ERK IR was equally low in normal and vehicle-injected eyes (Fig 2B and 2G). Intense P-ERK IR was seen in 2M6+ cells at 2 h after IRL1620 treatment (Fig 2C). Weaker IR was seen at 4 and 6 h (Fig 2D and 2E) and the IR was similar to normal by 24 h after the treatment (Fig 2F). Western blot analysis confirmed the P-ERK immunohistochemistry results (Fig 2H and 2I). The P-ERK levels were normalized to the expression levels of glyceraldehyde-3-phosphate dehydrogenase (GAPDH) (Fig 2I) or to total ERK (S4 Fig). Both methods of normalization gave similar results. Note that chicken ERK1/2 are displayed as one band on the western blot compared to two bands for mammalian ERK1/2 [32, 33]. A robust increase of P-ERK was seen 2 h after IRL1620 treatment compared to control. These results gave support to that *in vivo* stimulation of EDNRB by IRL1620 induced ERK1/2 activation in chicken retina including the Müller cells.

### Expression of endothelin receptors in chicken and human Müller cells in culture

**Fig 3:**
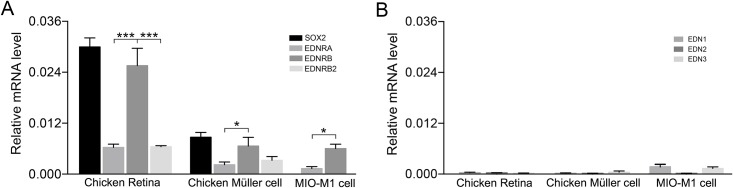
Relative mRNA levels of EDNs and EDNRs in E18 chicken retina, primary chicken Müller cells and the human MIO-M1 Müller cell line. qRT-PCR analysis of mRNA levels. Bar graphs showing the relative mRNA levels normalized to ß-actin for (A) EDNRA, EDNRB, EDNRB2 and SOX2, and for (B) EDN1, EDN2, EDN3 in chicken E18 retina (Chicken retina), primary chicken Müller cells (Chicken Müller cell) and the human MIO-M1 Müller cell line. Note that EDNRB2 is not found in human. For the MIO-M1 cells the relative mRNA levels of EDNRA and EDNRB are shown. Sox2 is included as an expression reference for the chicken cells. Bar graphs are mean ± SEM, n = 5 (*P < 0.01, ***P< 0.0001) analyzed by one-way ANOVA and Tukey’s post hoc test. Significance is only indicated for the comparisons: EDNRA-EDNRB, EDNRB-EDNRB2, EDN1-EDN2, and EDN2-EDN3.

We studied the expression of the endothelin receptors and their ligands in normal chicken retina, primary chicken Müller cells and in the human Müller cell line MIO-M1 by using qRT-PCR analysis. The levels of EDNRB mRNA were high relative to EDNRA and EDNRB2 in both chicken retina and primary Müller cell culture (Fig 3A). EDNRB mRNA expression was also higher in MIO-M1 cells than EDNRA mRNA expression (Fig 3A). Very low levels of the endothelin mRNA were seen in normal chicken retina, chicken primary, or human Müller cells (Fig 3B). The transcription factor SOX2 is expressed in chicken Müller cells [34] and was used as an expression reference. The results demonstrate that both chicken Müller cells and the human Müller cell-line MIO-M1 mainly express EDNRB. EDNRB2 was expressed at similarly low levels as that of EDNRA in chicken Müller cells.

### IRL1620 activates ERK1/2 MAPKases in primary chicken Müller cell and MIO-M1 cell cultures

**Fig 4:**
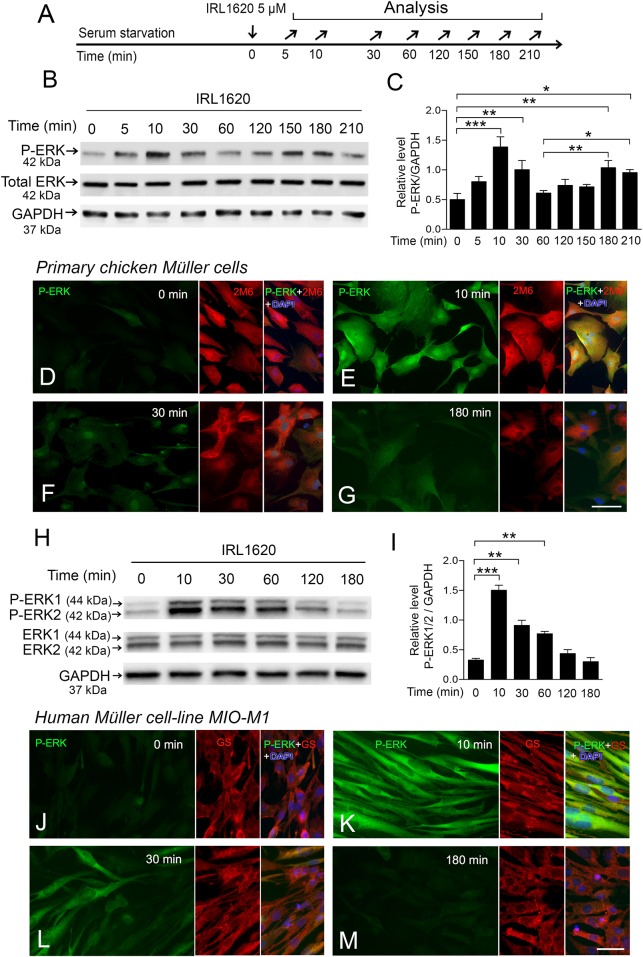
P-ERK1/2 in primary chicken Müller cells and the human MIO-M1 cell line after stimulation with IRL1620. (A) Experimental outline. (B-G) Primary chick Müller cells and (H-M) human MIO-M1 cells. Serum-starved cells were treated with 5 μM IRL1620, and analyzed at 5 min up to 210 min. (B) Western blot analysis of P-ERK levels in IRL1620-treated primary chick Müller cells. (C) Bar graph with densitometry of P-ERK levels normalized by GAPDH levels. Normalization to total ERK showed similar results (S5 Fig). Note that western blot analysis for ERK1/2 only shows one band in contrast to the two bands that are seen in mammals. (D–G) Fluorescence micrographs showing immunocytochemistry for P-ERK of IRL1620-treated primary chicken Müller cells at the indicated time points. 2M6 is a marker for chicken Müller cells. Cell nuclei were counter stained with DAPI. (H) Western blot analysis of P-ERK1/2 levels in IRL1620-treated MIO-M1 cells. (I) Bar graph with densitometry of P-ERK1/2 levels normalized by GAPDH levels. Normalization to total ERK1/2 showed similar results (S6 Fig). (J-M) Fluorescence micrographs showing immunocytochemistry for P-ERK in IRL1620-treated MIO-M1 cells at the indicated time points. Glutamine Synthetase (GS) is a Müller cell marker. 2M6 does not label human cells. Bar graphs are mean ± SEM, n = 3, (*P < 0.01, **P < 0.001, ***P < 0.0001) analyzed by one-way ANOVA and Tukey’s post hoc test. Significance is indicated for comparisons IRL1620 0 min and IRL1620 10, 30, 180 and 210 min; and IRL1620 60 min with IRL1620 180 and 210 min. Scale bar in (G and M) is 30 μm; valid also for (D-F and J-L).

Primary chicken Müller cells and the human cell-line MIO-M1 were stimulated by IRL1620 and phosphorylation of ERK1/2 signaling was studied by using western blot analysis and immunocytochemistry. We determined the dose-response of IRL1620 that gave an increased phosphorylation of ERK1/2 in the Müller cell cultures to 5μM (S3 Fig). To maintain a low basal level of P-ERK, the cells were serum-starved for 5 h (chicken cells) and 16 h (human cell line) respectively, treated with IRL1620 and analyzed at different time points (Fig 4A). The western blot analysis indicated two peaks with increased P-ERK levels (Fig 4B). The results were normalized to GAPDH (Fig 4B and 4C) or to total ERK levels (S5 Fig) that gave similar results. Densitometric analysis showed increased P-ERK levels within 5 min after IRL1620 treatment, peak levels at 10 min, with a decrease at 30 min. A second peak was seen by 180 min (Fig 4B and 4C). Immunocytochemistry showed strong cytoplasmic and nuclear P-ERK IR in the chicken Müller cells at 10 min (Fig 4E), which had decreased at 30 min (Fig 4F). Weak perinuclear P-ERK IR was seen 180 min after IRL1620 treatment (Fig 4E and 4G).

Western blot analysis of IRL1620-stimulated MIO-M1 cells showed an extended increase of P-ERK levels for 10–60 min (Fig 4H and 4I). Note the two ERK1/2 bands in human samples. The increase that was seen in chicken cells at 180 min was not seen in the MIO-M1 cells (Fig 4H and 4I). The 2M6 antibody does not stain human cells. Instead we used glutamine synthetase (GS) as a Müller cell marker [24] and immunocytochemistry showed that all cells in the MIO-M1 culture were GS+. Robust cytoplasmic P-ERK IR was seen at 10 min after IRL1620-treatment (Fig 4K) and moderate IR at 30 min (Fig 4L) in GS+ cells. We did not see any increased P-ERK IR at 180 min (Fig 4M). The data show that IRL1620 induced ERK1/2 activation in both primary chicken Müller cells and in human MIO-M1 cells, although with different temporal profiles of the ERK1/2 activation.

### EDNRB blocker BQ-788 inhibited ERK1/2 activation in Müller cells

**Fig 5:**
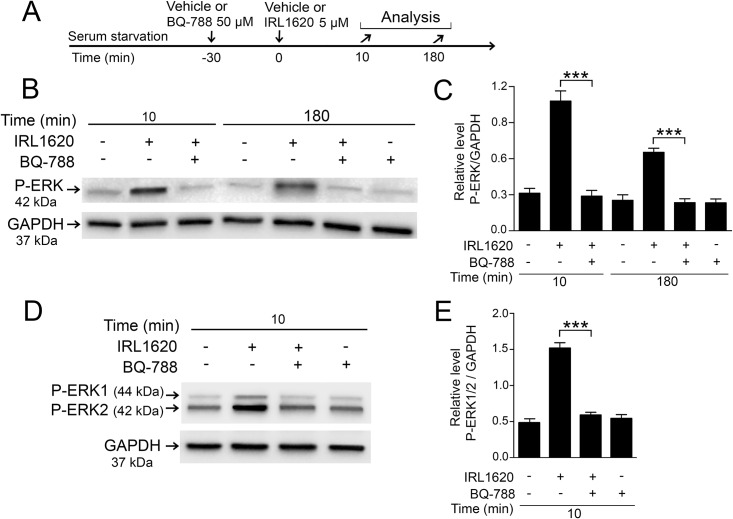
Effects of EDNRB blocker BQ-788 on IRL1620-induced P-ERK1/2 levels in primary chicken Müller cells and the human MIO-M1 cell line. Serum-starved primary chicken Müller cells and human MIO-M1 cells were pretreated with 50 μM EDNRB blocker BQ-788 or vehicle (control) for 30 min followed by 5 μM IRL1620 or vehicle for 10 and 180 min. (A) Experimental outline. (B, D) Representative western blot gels showing P-ERK levels in (B) primary chick Müller cells and (D) the human MIO-M1 cell line. (C, E) Bar graphs with densitometry of P-ERK levels normalized to GAPDH levels. Bar graphs are mean ± SEM, n = 3 (***P < 0.0001) analyzed by one-way ANOVA and Tukey’s post hoc test. Significance is indicated for the comparisons IRL1620 10 min-IRL1620+BQ-788 10 min and IRL1620 180 min-IRL1620+BQ-788 180 min.

To confirm that the activation of ERK1/2 by IRL1620 was due to stimulation of EDNRB signaling, we used a selective EDNRB blocker BQ-788 [35]. Serum starved primary chicken Müller cells and human MIO-M1 cells were pre-treated with BQ-788 and then treated with IRL1620 (Fig 5A). Western blot with densitometric analyses showed that BQ-788 treatment reduced the P-ERK levels to control levels both at the 10 and 180 min time points in primary chicken Müller cells (Fig 5B and 5C). Because the MIO-M1 cells did not show any increased P-ERK levels at the 180 min time point, only the 10 min time point was tested. BQ-788 attenuated the IRL1620-induced P-ERK1/2 increase (Fig 5D and 5E). Cells treated only with BQ-788 did not alter the basal P-ERK levels (Fig 5B and 5C). The data support that IRL160 induces ERK1/2 activation by stimulation of EDNRB signaling.

### IRL1620-induced ERK1/2 activation engage EGFRs in primary chicken and human Müller cell cultures

**Fig 6:**
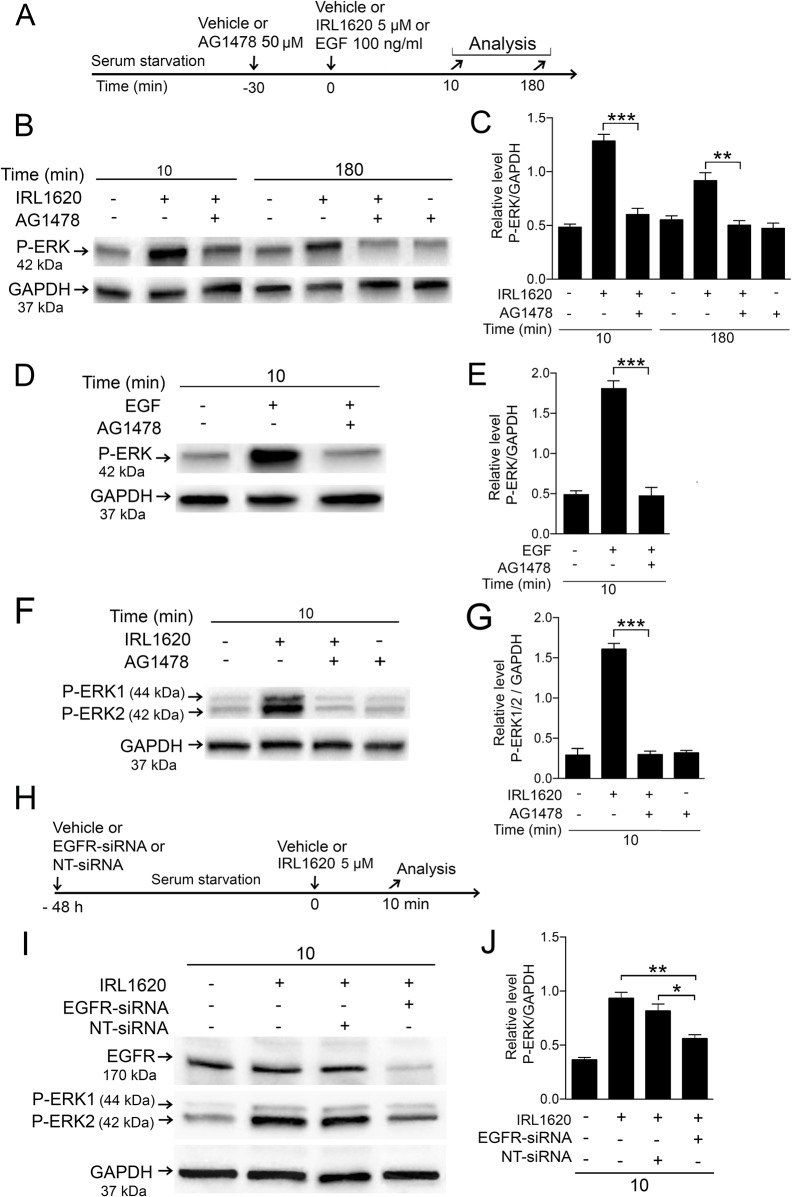
Effects of EGFR kinase inhibitor AG1478 or EGFR-siRNA on IRL1620-induced P-ERK1/2 levels in Müller cells. Serum-starved primary chicken Müller cells and human MIO-M1 cells pretreated with 50 μM AG1478 or control (vehicle) for 30 min followed by treatment with 5 μM IRL1620 or vehicle for 10 and 180 min of chicken Müller cells, and for 10 min of human MIO-M1 cells. (A) Experimental outline. (B-G) Western blot analyses of P-ERK levels in (B, C) chicken Müller cells treated with IRL1620 and AG1478, and (D, E) EGF + AG1478. (C, E) Bar graphs with densitometry of P-ERK levels normalized by GAPDH levels. (F) Western blot analysis of P-ERK1/2 levels in human MIO-M1 cells treated with IRL1620 + AG1478. Note that western blot analysis for ERK1/2 shows two bands in human compared to one band in chicken. (G) Bar graph with densitometry of P-ERK1/2 levels normalized to GAPDH levels. (H) Human MIO-M1 cells transfected with EGFR-siRNA or non-targeted siRNA (NT-siRNA) in absence of serum for 48 h followed by treatment with 5 μM IRL1620 or vehicle for 10 min. (I) Western blot analysis of P-ERK 1/2 levels in human MIO-M1 cells treated with IRL1620+EGFR-siRNA or IRL1620+NT-siRNA. (J) Bar graph with densitometry of P-ERK1/2 levels normalized to GAPDH levels. Bar graphs are mean ±SEM, n = 3 (*P < 0.01, **P < 0.001, ***P < 0.0001) analyzed by one-way ANOVA and Tukey’s post hoc test. Significance is only indicated for the comparisons: IRL1620 10 min-IRL1620+AG1478 10 min, IRL1620 180 min-IRL1620+AG1478 180 min, IRL1620 10 min-IRL1620+NT-siRNA 10 min and IRL1620 10 min-IRL1620+EGFR-siRNA 10 min.

Endothelin receptors are GPCRs [9] and have been shown to transactivate EGFRs in different cell types [36]. To assess whether the EDNRB-triggered P-ERK involve EGFRs in the Müller cells, we studied the effect of AG1478, a potent EGFR kinase inhibitor [37] and siRNA knock-down of EGFR expression on the IRL1620-induced ERK1/2 activation. As a control we studied EGF-induced ERK1/2 activation in the primary chicken Müller cells. Serum starved Müller cells were treated with EGFR-blocker AG1478 and after 30 min the cells were treated with IRL1620 or EGF (Fig 6A). The chicken cells were tested at the 10 min and 180 min time points and the human cells at the 10 min time point. Western blot with densitometric analysis was used to quantify the effects (Fig 6B–6G). Blocking of the EGFR kinase by AG1478 reduced IRL1620-induced P-ERK to basal levels in the chicken cells at both the early 10 min and at the late 180 min time points (Fig 6B and 6C). We have previously shown that EGF triggers a robust P-ERK-response in chicken Müller cells by 10 min [23] and the result showed that AG1478 inhibited this EGF-induced ERK1/2 activation in primary chicken Müller cells (Fig 6D and 6E). AG1478 treatment also blocked IRL1620-induced P-ERK1/2 in MIO-M1 cells at the 10 min time point (Fig 6F and 6G). AG1478-only treatment neither altered the basal P-ERK levels in chicken nor in the human cells (Fig 6B and 6F). MIO-M1 cells were transfected and treated with EGFR-siRNA or non-targeted siRNA control for 48 h followed by stimulation with IRL1620 for 10 min (Fig 6H). Western blot analysis showed that the EGFR-siRNA specifically reduced the EGFR levels without affecting the GAPDH levels (Fig 6I and 6J). Consistent with the reduced EGFR level the EGFR-siRNA significantly reduced the P-ERK1/2 levels compared to non-transfected or non-target siRNA control (Fig 6I and 6J). The capacity of an EGFR blocker and an EGFR-siRNA to attenuate IRL1620-induced P-ERK1/2 indicate that the EGFR is engaged in the EDNRB-response in Müller cells.

**Fig 7:**
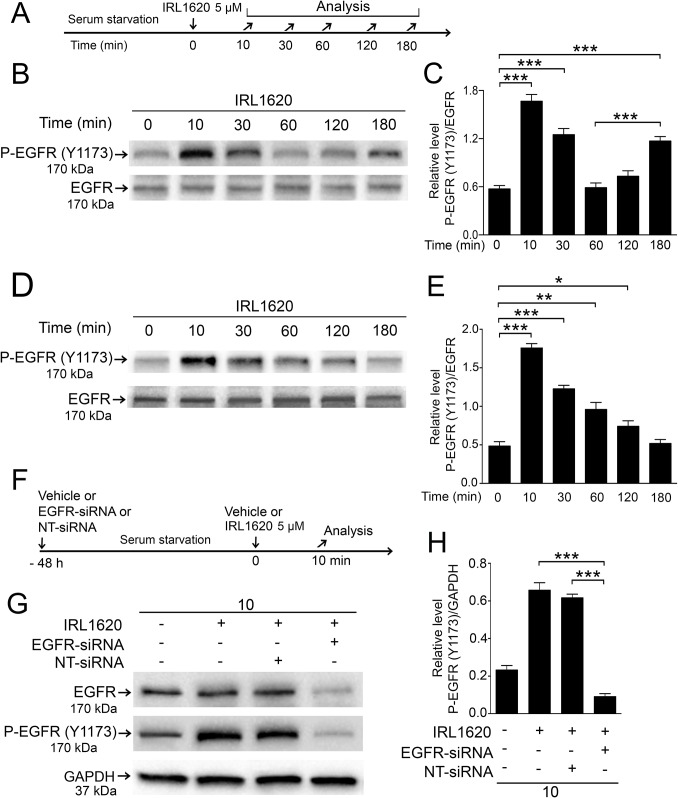
Effects of IRL1620 on Y1173-EGFR phosphorylation and EGFR-siRNA on IRL1620-induced Y1173-EGFR phosphorylation in Müller cells. Serum-starved cells treated with 5 μM IRL1620 and P-EGFR was analyzed at different time points from 10 min to 180 min. (A) Experimental outline. Western blot analysis of P-(Y1173)-EGFR in (B) primary chicken Müller cells and (D) the human MIO-M1 cell line. Bar graphs with densitometry of P-EGFR levels normalized by total EGFR levels in (C) primary chicken Müller cells and (E) the human MIO-M1 cell line. (F) MIO-M1 human Müller cells transfected with EGFR-siRNA or non-targeted siRNA (NT-siRNA) in absence of serum for 48 h followed by treatment with 5 μM IRL1620 or vehicle for 10 min. (G) Western blot analysis of P-(Y1173)-EGFR levels in human MIO-M1 cells treated with IRL1620+EGFR-siRNA or IRL1620+NT-siRNA. (H) Bar graph with densitometry of P-(Y1173)-EGFR levels normalized to GAPDH levels. Bar graphs are mean ± SEM, n = 3 (*P < 0.01, **P < 0.001, ***P < 0.0001) analyzed by one-way ANOVA and Tukey’s post hoc test. Significance is only indicated for IRL1620 0 min with IRL1620 10, 30 and 180 min; IRL1620 60 min with IRL1620 180 min; IRL1620 10 min with IRL1620+NT-siRNA 10 min and IRL1620 10 min with IRL1620+EGFR-siRNA 10 min.

We studied the phosphorylation of EGFR on Y1173 after IRL1620 treatment. Phosphorylation of Y1173 occurs as a result of ligand-dependent activation during autophosphorylation of the EGFR receptor. Y1173 autophosphorylation allows interaction of adaptor proteins Grb2 and Shc with the receptor and mediate Ras-activated MAPK signaling, including ERK1/2 [38, 39]. Serum-starved chicken and human cells were treated with IRL1620 and analyzed at time points from 10 min to 180 min (Fig 7A). Densitometric analyses were performed to quantify the P-EGFR (Y1173) levels after IRL1620 treatment (Fig 7B–7E). In primary chicken Müller cells a similar pattern as for the P-ERK was seen for the P-EGFR (Y1173), with peak levels at 10 min and 180 min (Fig 6B and 6C) and in the human MIO-M1 cells a broader peak of P-EGFR (Y1173) was seen at 10 min that gradually decreased to control levels by 180 min (Fig 7D and 7E). The siRNA knock down of EGFR expression in MIO-M1 cells reduced the phosphorylation of EGFR Y1173 after IRL1620 treatment (Fig 7F and 7H). The EGFR-siRNA knock down showed reduced P-EGFR (Y1173) levels compared to non-transfected or non-targeted siRNA control (Fig 7G and 7H). The results are consistent with that IRL1620-induced ERK1/2 activation engages EGFRs in chicken Müller cells and in the human MIO-M1 cells.

### IRL1620-induced ERK1/2 activation requires cytosolic Src-kinase activity

**Fig 8:**
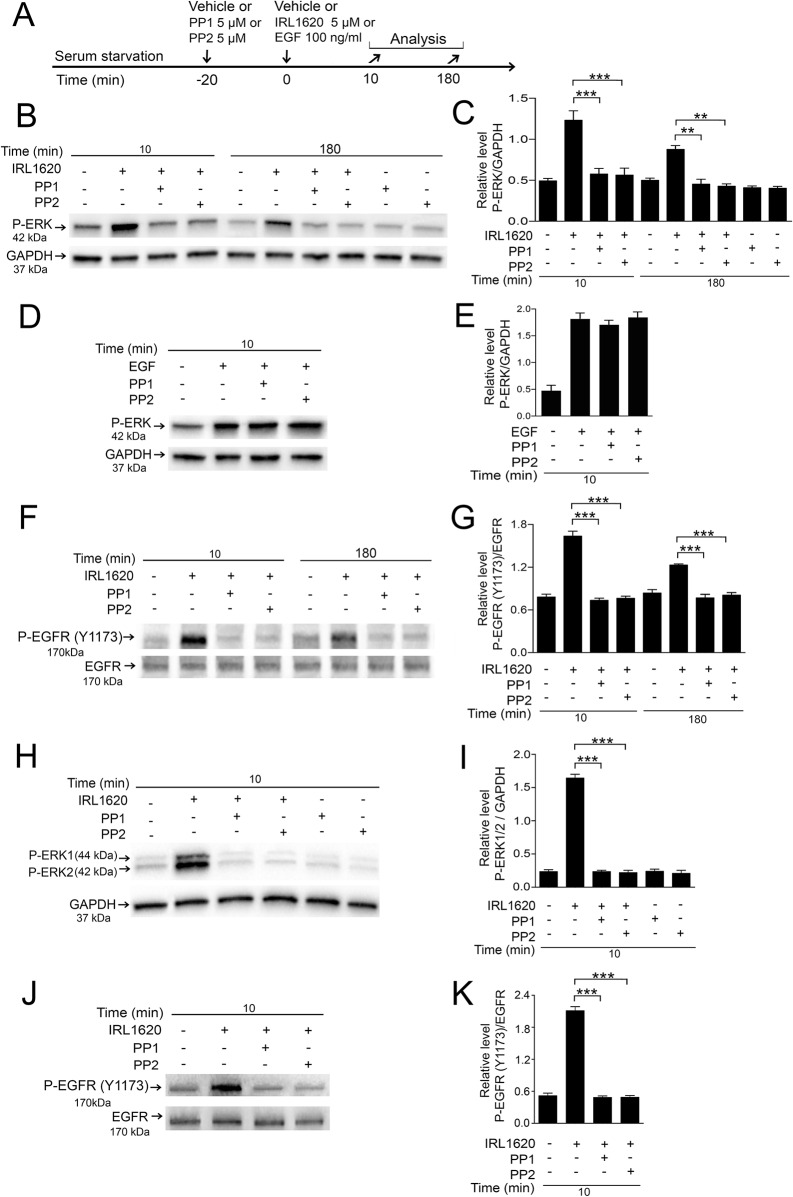
Effects of Src-kinase inhibitors PP1 and PP2 on IRL1620-induced P-ERK1/2 and P-EGFR (Y1173) in primary chicken Müller cells and the human MIO-M1 cell line. Serum-starved primary chicken Müller cells and human MIO-M1 cells were pretreated with 5 μM PP1 or 5 μM PP2, or control (vehicle) for 20 min followed by treatment with 5 μM IRL1620 or vehicles and analyses. Western blot was used to analyze ERK-signaling with the different treatments. (A) Experimental outline. The MIO-M1 cells were only analyzed at 10 min because the peak at 180 min is not present. Western blot analyses of (B-E) P-ERK levels and (F, G) P-EGFR (Y1173) in chicken Müller cells. (C, E) Bar graphs with densitometry of P-ERK levels normalized by GAPDH and (G) P-EGFR (Y1173) levels normalized to total EGFR levels. Western blot analyses of (H) P-ERK1/2 levels and (J) P-EGFR (Y1173) levels in human MIO-M1 cells. Bar graphs with densitometry of (I) P-ERK1/2 levels normalized by GAPDH and (K) P-EGFR (Y1173) levels normalized to total EGFR levels. Bar graphs are mean ±SEM, n = 3 (***P < 0.0001) analyzed by one-way ANOVA and Tukey’s post hoc test. Significance is only indicated for the comparisons: IRL1620 10 min-IRL1620+PP1 10 min, IRL1620 10 min-IRL1620+PP2 10 min, IRL1620 180 min-IRL1620+PP1 180 min, and IRL1620 180 min-IRL1620+PP2 180 min.

Cytosolic Src is a non-receptor tyrosine kinase that is known to play a role in GPCR-mediated ERK1/2 activation via transactivation of EGFR [40, 41]. To assess whether IRL1620-induced ERK1/2 activation is Src-kinase dependent in Müller cells, we studied the effect of Src-kinases inhibitors; PP1 and PP2, on IRL1620-induced ERK1/2 and Y1173 EGFR activation in the chicken and human cells. We also tested whether the Src-kinase inhibitor could block the EGF-induced ERK1/2 activation in the cells. Serum starved Müller cells were pretreated with PP1 or PP2 and after 20 min IRL1620 or EGF were added to the cultures (Fig 8A). Western blot with densitometric analyses were used to study the effects of PP1 and PP2 on ERK1/2 activation (Fig 8B–8E, 8H and 8I) and on EGFR (Y1173) activation (Fig 8F, 8G, 8J and 8K). The 10 min and 180 min time points were tested for chicken cells while only the 10 min time point was tested for the MIO-M1 cells for reasons already discussed. PP1 or PP2-treatment reduced P-ERK to control levels in primary chicken Müller cells at both the early 10 min and the late 180 min time points (Fig 8B and 8C). There was no effect on the EGF-induced P-ERK by PP1 or PP2 treatments (Fig 8D and 8E). PP1 or PP2 reduced the P-ERK1/2 levels to basal levels also in the human MIO-M1 cells (Fig 8H and 8I). Treatment with PP1 or PP2 did not alter the basal P-ERK levels (Fig 8B, 8C, 8H and 8I). These results are consistent with that cytosolic Src-kinases are involved in IRL1620-induced ERK1/2 activation both in primary chicken Müller cells and in the human MIO-M1 cells. P-EGFR (Y1173) levels were reduced by PP1 and PP2 treatments at the early and late phases in the chicken Müller cells (Fig 8F and 8G). PP1 and PP2 treatment of the human cells reduced P-EGFR (Y1173) levels (Fig 8J and 8K). The data indicate that IRL1620-induced EGFR and ERK1/2-activation is Src-kinase dependent both in the primary chicken Müller cells and in the human MIO-M1 cells.

### Matrix metalloproteinase (MMP) inhibitor GM6001 attenuates IRL1620-induced ERK1/2 activation in Müller cell cultures

**Fig 9:**
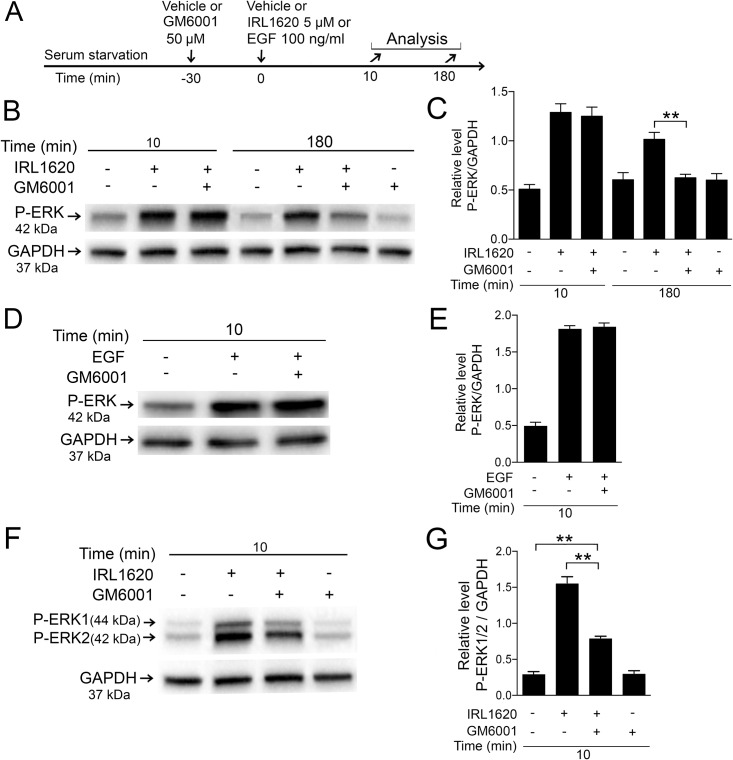
Effect of MMPs inhibitor GM6001 on IRL1620- induced P-ERK1/2 in primary chicken Müller cells and the human MIO-M1 cell line. Serum-starved primary chicken Müller cells and human MIO-M1 cells pretreated with 50 μM GM6001 or control (vehicle) for 30 min followed by treatment with 5 μM IRL1620 or vehicles were analyzed. (A) Experimental outline. The MIO-M1 cells were only analyzed at 10 min because the peak at 180 min is not present. Western blot analyses of P-ERK levels in (B) chicken Müller cells treated with IRL1620 and/or GM6001 and (D) EGF and/or GM6001. EGF-treatment was only analyzed at time point 10 min. (C, E) Bar graphs with densitometry of P-ERK levels normalized by GAPDH levels. Western blot analysis of P-ERK1/2 levels in (F, G) human MIO-M1 cells treated with IRL1620 and/or GM6001. (G) Bar graph with densitometry of P-ERK1/2 levels normalized by GAPDH levels. Bar graphs are mean ±SEM, n = 3 (**P < 0.001) analyzed by one-way ANOVA and Tukey’s post hoc test. Significance is only indicated for the comparisons: IRL1620 10 min-IRL1620+GM6001 10 min, and IRL1620 180 min-IRL1620+GM6001 180 min.

It has been shown that MMPs are involved in GPCR-mediated transactivation of EGFR in different cell types [42, 43]. Activation of MMPs can release heparin binding-EGF (HB-EGF) by cleaving membrane bound pro-HB-EGF. HB-EGF activates EGFRs in an autocrine mode and subsequently induces ERK1/2 activation [38]. To test whether ERK1/2 activation by IRL1620 in primary chicken and human Müller cells involves MMP activity, we analyzed the effect of the broad-spectrum MMP inhibitor GM6001 [44] on IRL1620-induced P-ERK1/2. Serum-starved Müller cells were pretreatment with GM6001 for 30 min before the addition of IRL1620 or EGF (Fig 9A). Western blot with densitometric analysis was used to study the effect of GM6001 (Fig 9B–9G). Interestingly, in the chicken Müller cells, GM6001 attenuated the late 180 min time point-increase of P-ERK but not the early 10 min phase of P-ERK, pointing to a role of MMPs in ERK1/2 activation in Müller cells by IRL1620 treatment (Fig 9B and 9C). GM6001 pretreatment also attenuated the IRL1620-induced P-ERK in the MIO-M1 cells (Fig 9F and 9G). Treatment with GM6001 before EGF did not alter P-ERK levels (Fig 9D and 9E). The basal P-ERK levels were not altered with GM6001-only treatment (Fig 9B and 9G). The data indicates that MMPs contribute to ligand-dependent ERK1/2 activation both in primary chicken Müller cells and in human MIO-M1 cells.

**Fig 10:**
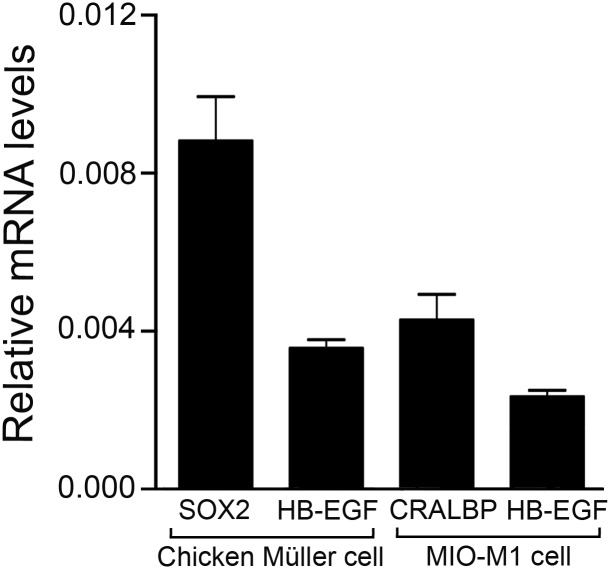
Expression of HB-EGF in chicken Müller cells and in human MIO-M1 cell line. QRT-PCR analysis of heparin binding epidermal growth factor (HB-EGF), transcription factor SOX2 and cellular retinaldehyde-binding protein (CRALBP) mRNA levels in primary chicken Müller cell and in human MIO-M1 cell cultures. SOX2 and CRALBP were included as expression references known to be expressed in Müller cells. Bar graph showing the relative mRNA levels in relation to β-actin mRNA levels. Bar graph is mean ±SEM, n > 5.

The HB-EGF is one of the endogenous EGFR ligands that can mediate EGFR activation. We analyzed the expression of HB-EGF in primary chicken Müller cell and in MIO-M1 cells by qRT-PCR analysis and HB-EGF mRNA expression was seen both in primary chicken Müller cell and in the MIO-M1 cell culture (Fig 10). Sox2 is expressed in chicken Müller cells and the cellular retinaldehyde-binding protein (CRALBP) is expressed in MIO-M1 cells [45] and were used as expression level references.

## Discussion

**Fig 11:**
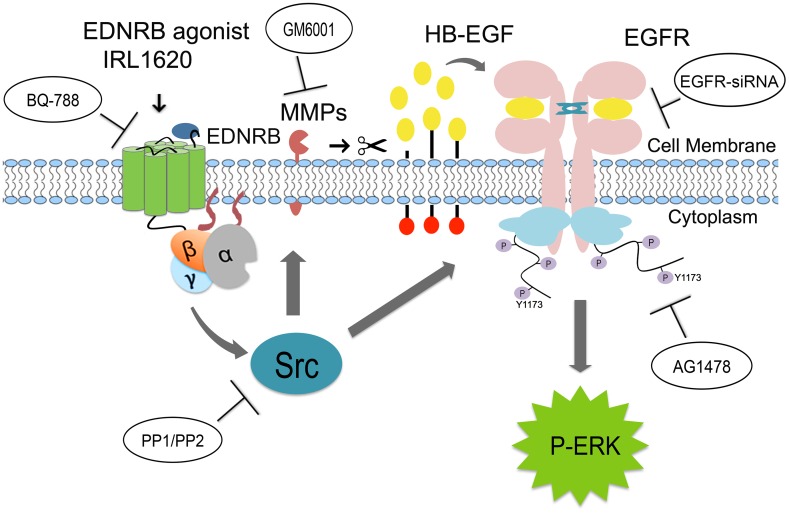
Illustration summarizing the EDNR signaling leading to transactivation of EGFR in Müller cells. Diagram showing the signal transduction events and inhibitors with names within ellipses that were used to block the signaling steps. Stimulation of EDNRB in Müller cells with EDNRB-agonist IRL1620 that leads to activation of cytosolic Src kinase. The activated Src kinase triggers matrix-metalloproteinase (MMP), whose catalytic activity leads to release of membrane-bound heparin-binding epidermal growth factor (HB-EGFR) and consequently causes ligand-dependent transactivation of the epidermal growth factor receptor (EGFR). Activated Src kinase may also trigger ligand-independent EGFR phosphorylation of tyrosine residue (Y1173). This transactivation leads to MAPK/ERK signaling in Müller cells that could be blocked by the EDNRB antagonist BQ-788, Src-kinase inhibitors PP1 and PP2), EGFR-inhibitor AG1478, EGFR-small interfering RNA (EGFR-siRNA) or by the inhibitor GM6001, which inhibits extracellular matrix metalloproteinases.

In this study, we first confirmed that the expression of the EDNs and EDNRs are injury-induced. A robust increase of EDN1, EDN2 and EDNRB mRNA expression was seen in the excitotoxically injured retina and EDNRB expression was seen in chicken Müller cells and the MIO-M1 Müller cell line in culture. Stimulation of EDNRBs with the agonist IRL1620, activated the MAPK signal transduction pathway by increasing P-ERK1/2 levels in Müller cells in retina *in vivo* as well as in culture. P-ERK signaling regulates the injury-response of the Müller cells either to dedifferentiate, proliferate and form retinal progenitor cells or to maintain the homeostatic functions of Müller cells. The P-ERK response after IRL1620 stimulation was used to study the EDNR MAPK signal transduction pathway in Müller cells and the results showed that the IRL1620-induced P-ERK-signal is dependent on the Src-kinase and involves both ligand-dependent and ligand-independent activation of EGFRs. The data are summarized in Fig 11 in a schematic diagram indicating the signal pathways from the EDNR via Src and MMPs to the EGFR and P-ERK as well as the actions of the inhibitors and reagents that are used to probe the pathway in this study. The results are indicative of EDNR mediated transactivation of EGFR on Müller cells.

The EDNs are pleiotropic and produce several different and sometimes opposing effects in different cells and physiological situations. This pleiotropy may be explained by the broad spectrum of signal transduction effectors that are activated by the EDNRs. Our results are consistent with data showing that EDN2 may serve as a signaling agent in retina to Müller cells and that EDN2 may induce genes associated with EGFR signaling and reactive gliosis in retinal Müller cells [3, 17, 46]. We find similar effects of the EDNR agonist IRL1620 in the chicken retina *in vivo*, on primary chicken Müller cells *in vitro* as well as on the human Müller MIO-M1 cell line, indicating that the signal mediator role for EDNs to Müller cells is a conserved feature. Neuroprotective effects of EDN2 and EDNRB, which has been seen during photoreceptor degenerations [17, 18] may relate to the capacity of EDNRs to modulate the injury-response by Müller cells in a similar way as has been seen by α2-adrenergic receptor agonists [47]. Some of the adverse effects by EDN1 in retina have been suggested to be associated with the vasoactive properties. The retinal blood flow via the ophthalmic artery is regulated by EDN1 [48] and over expression of EDN1 in endothelial cells cause progressive retinal degeneration [16]. EDN1 has also been suggested to act directly on retinal ganglion cells to induce apoptosis [11, 49].

The expression of EDNRB and EDNs is upregulated after several different CNS injuries [3, 18, 50]. In this study we show that excitotoxic retinal injury induced the expression of EDNRB and EDNs, giving support to the notion that up-regulation of EDNRB and EDNs is a common phenomenon after injury. Stimulation of EDNRB on Müller cells with IRL1620 resulted in a robust activation of ERK1/2 and similar effects have been seen in rat astrocytes [51, 52]. The specificity of IRL1620-induced ERK1/2 activation was confirmed by the ability of the EDNRB-specific antagonist BQ-788 to attenuate the increase of P-ERK1/2 (Fig 5).

Our results showing that IRL1620-stimulation of EDNRB activates EGFRs on Müller cells are consistent with that EDN1 transactivated EGFRs on Rat1 cells [36] as well as on vascular smooth muscle cells [7, 22]. The specificity of EDNRB-mediated activation of EGFR was shown by the ability of the EGFR-kinase inhibitor AG1478 and EGFR siRNAs to reduce phosphorylation of both the EGFR and ERK1/2. Our data show that IRL1620-induced activation of EGFR and ERK1/2 is dependent on Src-kinase activity. In PC12 cells epinephrine-induced ERK1/2 activation requires Src-kinase activity but has both an EGFR-dependent and an independent component [43]. The Src-kinase blockers, PP1 and PP2 abrogated the IRL1620-induced ERK1/2 activation but not the EGF-induced ERK1/2 activation, suggesting that ligand-activated EGFR signaling *per se* in Müller cells does not require Src-kinase activity. Src-kinases have been shown to associate with the catalytic domain of EGFR and activate EGFR in epithelial and fibroblast cells in a ligand-independent way [53, 54]. In line with these findings, Src-kinase can directly activate the EGFR kinase and consequently activate ERK1/2 in Müller cells (Fig 11). Stimulation of EDNRB in Müller cells resulted in phosphorylation of tyrosine residue 1173 of the EGFR, which is one of the major autophosphorylation site that allows binding of GRB2, SOS and SHC adapter proteins to EGFR and that mediates Ras-Raf activated MAPK/ERK signaling [39]. This autophosphorylation event is indicative of ligand-dependent activation of EGFR and supports a mechanism that involves an autocrine mode of action on Müller cells with transactivation of EGFRs.

Plasma membrane associated extracellular MMPs have been shown to be key effectors of GPCR-mediated EGFR transactivation [36]. Activated MMPs proteolytically release membrane bound pro-HB-EGF or TGFα that activate EGFRs in an auto- or paracrine mode of action [43]. Src-kinases are involved in MMP activation by interaction with proline-rich Src-homology domain on the intracellular portion of the MMP proteins [55]. Based on the MMP inhibition studies using GM6001 that reduced P-ERK1/2 levels during the late phase at 180 min but not during the early phase at 10 min, we concluded that both ligand-dependent and ligand-independent transactivation of EGFR occur in chicken Müller cells. These results are consistent with previous data from vascular smooth muscle cells in which the N-terminal domain of EDNRB is important for a biphasic ERK1/2 activation by MMP-2 mediated-ligand-dependent transactivation of EGFRs [7]. In the human MIO-M1 cells, inhibition of MMPs with GM6001 also resulted in reduced P-ERK1/2 levels, indicating that a ligand-dependent mechanism for EGFR transactivation is mainly in effect. However, a complete attenuation of the activation of ERK1/2 could not be seen, indicating that both EGFR ligand-dependent and ligand-independent transactivation of EGFR occurs simultaneously in the MIO-M1 cells. The role for this heterogeneity between the chicken and human cells may be species related and may reflect differences in the intrinsic cellular properties. It is well known that the regenerative capacity of mammalian Müller cells is lower in mammals than in birds. Chicken Müller cells have the capacity to dedifferentiate and proliferate, which are the initial steps in regeneration whereas the Human Müller cells do not have that capacity to a full extent. This difference in capacity may be associated with the temporal differences of ERK signaling shown in our data.

The chicken Müller cells and human MIO-M1 cells express HB-EGF (Fig 10) but it remains to be proven that HB-EGF is the endogenous ligand involved in the transactivation of EGFR. HB-EGF is required for Müller cell proliferation and dedifferentiation into progenitor cells both in the injured and uninjured retina [21]. In PC12 cells, the intensity and duration of ERK1/2 activation determines the cellular response; either to proliferate or differentiate [56, 57]. In chicken, excitotoxic retinal injury induces a sustained ERK1/2 activation in Müller cells that leads to proliferation and dedifferentiation [32]. Moreover, normal ERK1/2 signaling in retina has also been shown to have protective roles during excitotoxic injury [58]. Previously we have shown that transient ERK1/2 activation by alpha 2-adrenerigc receptor agonist modulates the Müller cell-response to injury by attenuating injury-induced ERK1/2 levels using a negative feedback regulation in Müller cells [47]. Such data are in line with and open up for the possibility to pharmacologically modulate the Müller cell response after injury by targeting EGFR transactivation. It remains to be studied what are the effects of the transactivation in context of retinal damage or neuroprotection of retina.

## Conclusion

Our data show that both chicken and human Müller cells express EDNRB and that stimulation of the Müller cells by the agonist IRL1620 leads to Src-kinase mediated ERK1/2 activation that engages both ligand-dependent and ligand-independent EGFR transactivation. These results imply that injury-induced endothelin-signaling in retina activates or modulates the Müller cell response by EDNR-triggered transactivation of EGFR signaling.

## Supporting Information

S1 Fig qRT-PCR primer amplification and melting graphs of *β-Actin*, *EDNRs*, *EDNs*, *HB-EGF* and *CRALBP* in chicken and human Müller cells. (TIF)

S2 Fig qRT-PCR primer amplification and melting graphs of *EDNRs* and *EDNs* 24 h after excitotoxic injury in the chicken retina. (TIF)

S3 Fig Western blot analysis for P-ERK in E18 chicken retina activated by IRL1620, normalized to total ERK levels. (TIF)

S4 Fig Western blot analysis of P-ERK levels in E18 chicken retina activated by IRL1620, normalized to total ERK levels. (TIF)

S5 Fig Western blot analysis of P-ERK levels in chick primary Müller cells activated by IRL1620, normalized to total ERK levels. (TIF)

S6 Fig Western blot analysis of P-ERK1/2 levels in human Müller cells activated by IRL1620, normalized to normal ERK1/2 levels. (TIF)

S1 Table List of reagents and inhibitors. (PDF)

S2 Table List of primary and secondary antibodies. (PDF)

S3 Table List of qRT-PCR primer sequences. (PDF)
