# Supplementary figures and images for: Endothelin B Receptors on Primary Chicken Müller Cells and the Human MIO-M1 Müller Cell Line Activate ERK Signaling via Transactivation of Epidermal Growth Factor Receptors
Source: PLoS One. 2016 Dec 8;11(12):e0167778. doi: 10.1371/journal.pone.0167778 (PMC5145189; doi:10.1371/journal.pone.0167778)

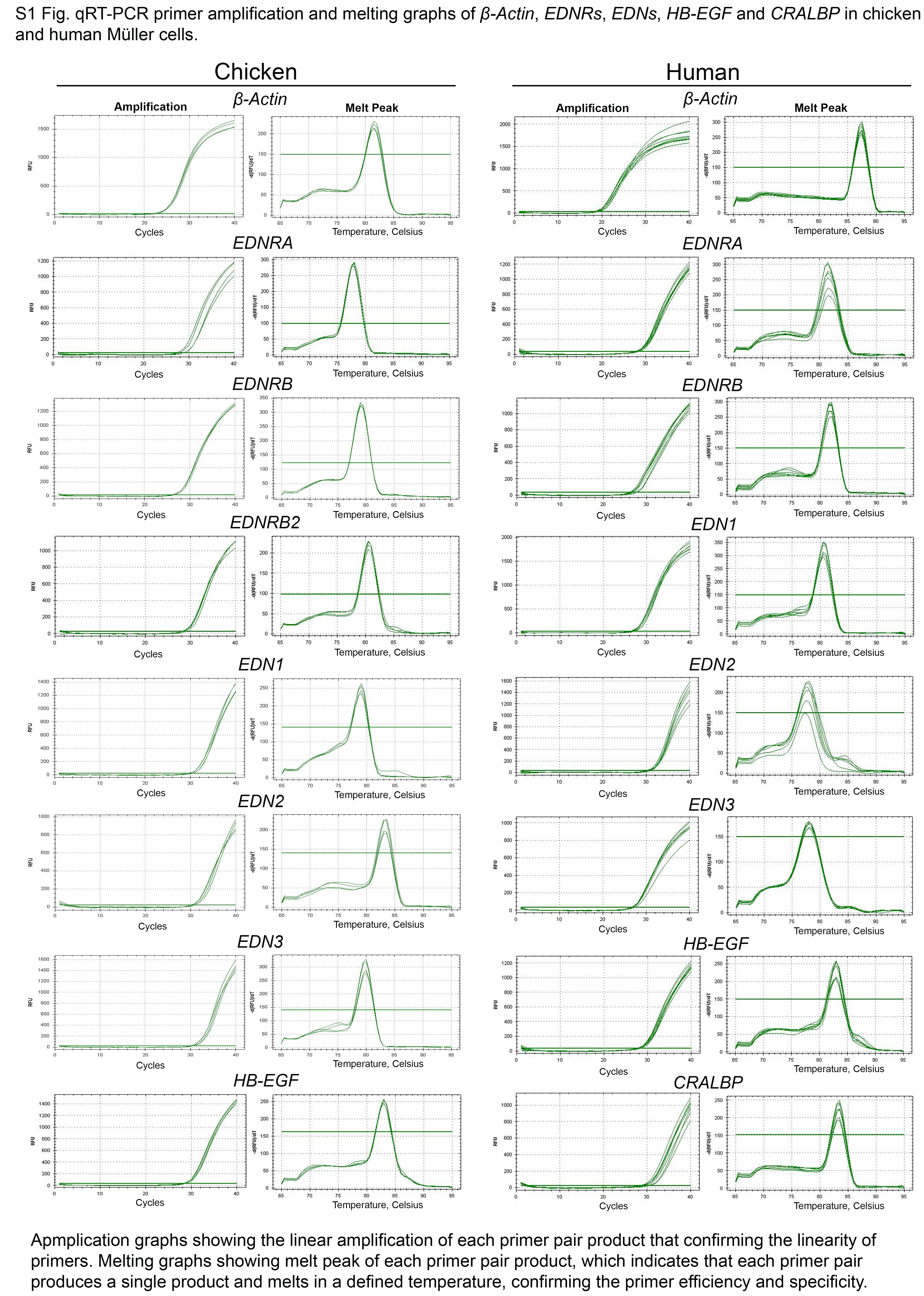

Supplement: S1 Fig — (TIF) [file pone.0167778.s001.tif]

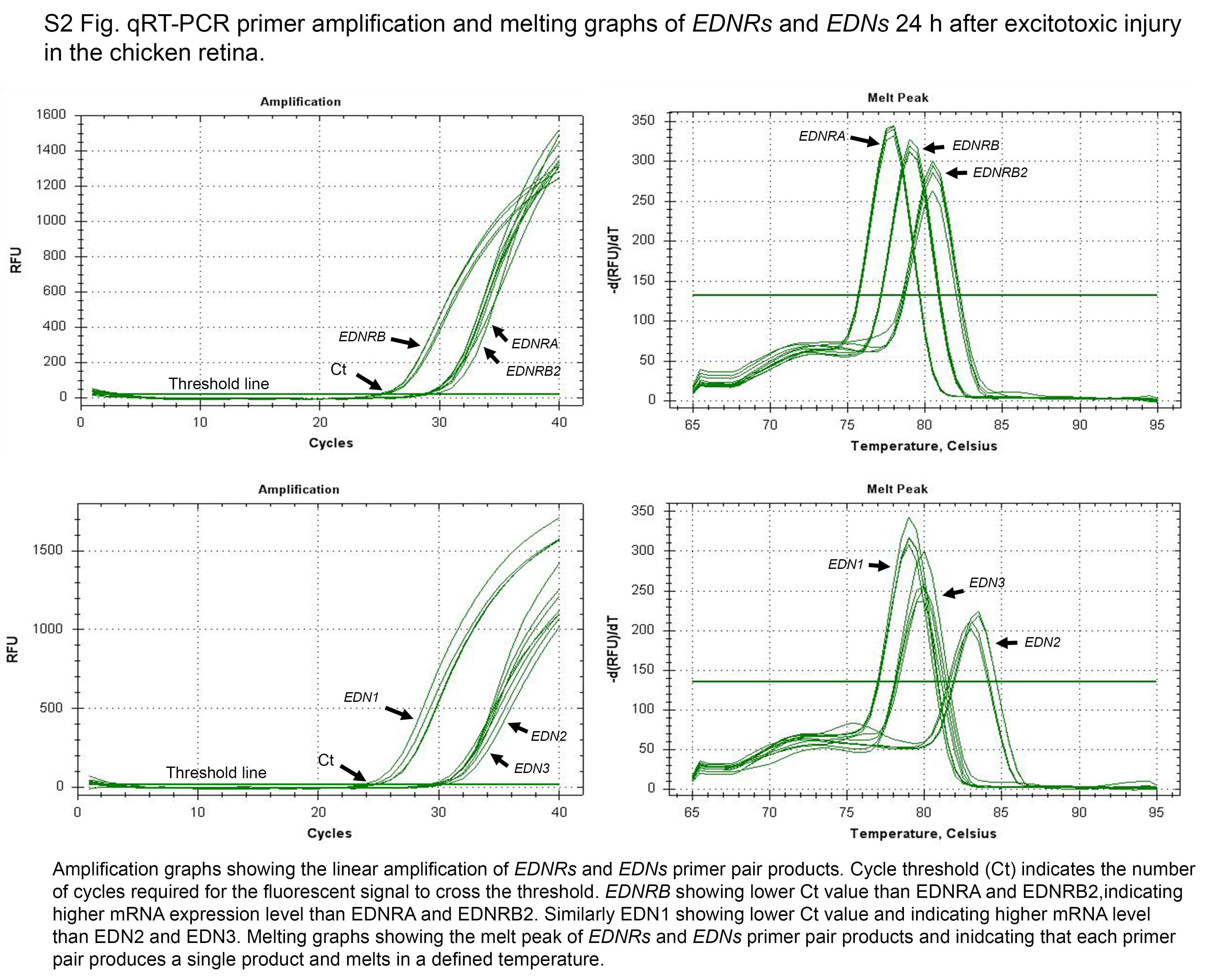

Supplement: S2 Fig — (TIF) [file pone.0167778.s002.tif]

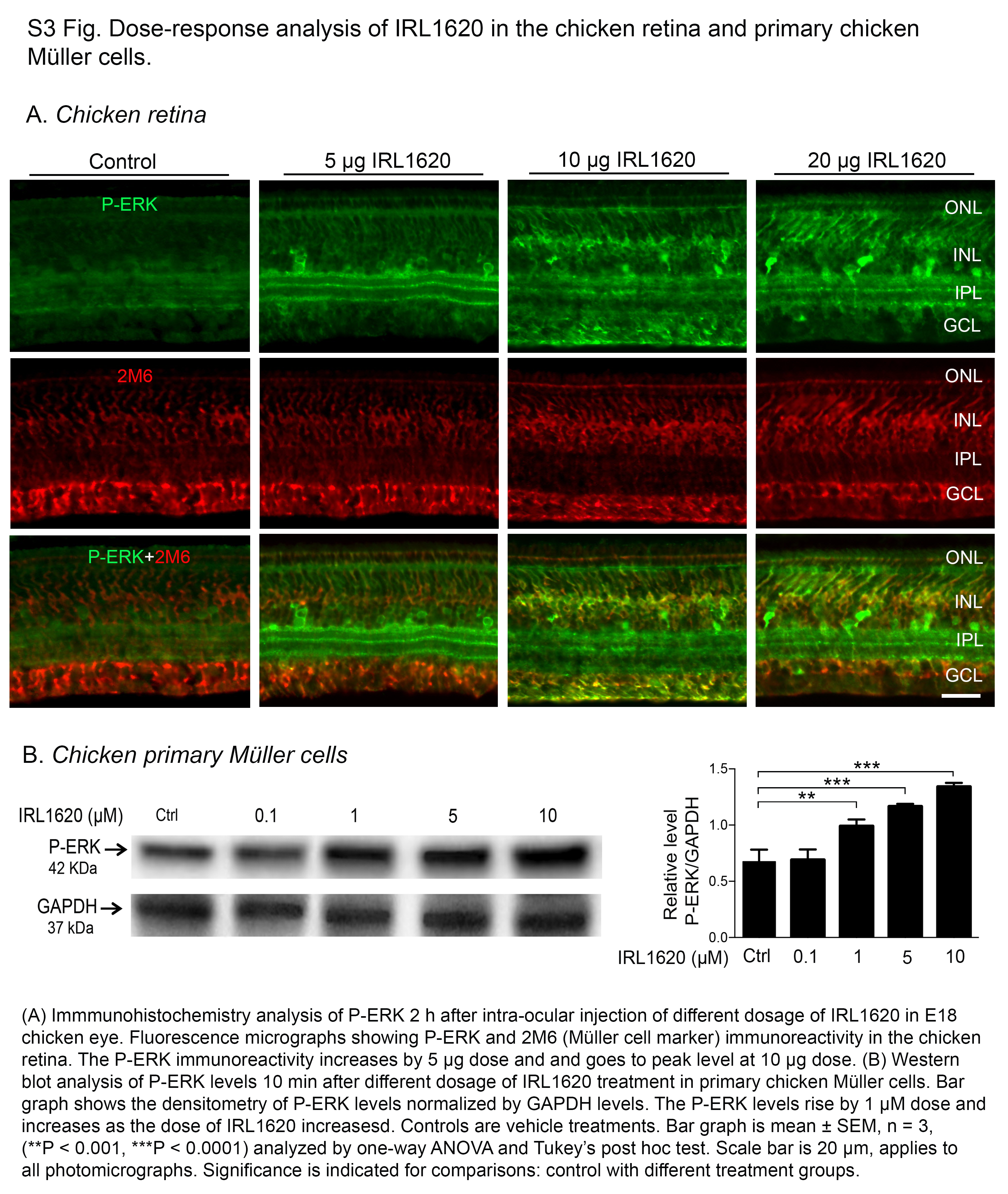

Supplement: S3 Fig — (TIF) [file pone.0167778.s003.tif]

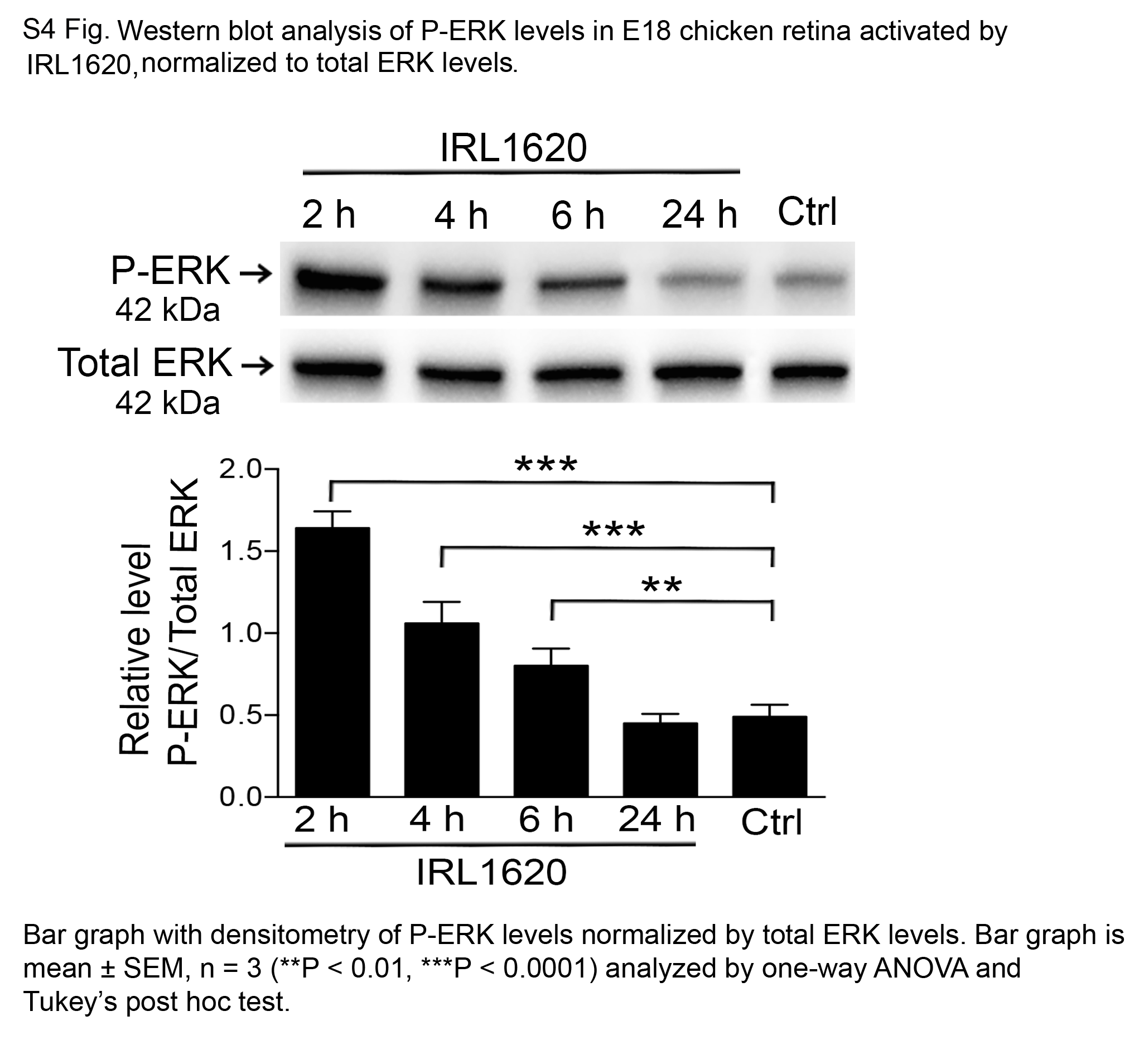

Supplement: S4 Fig — (TIF) [file pone.0167778.s004.tif]

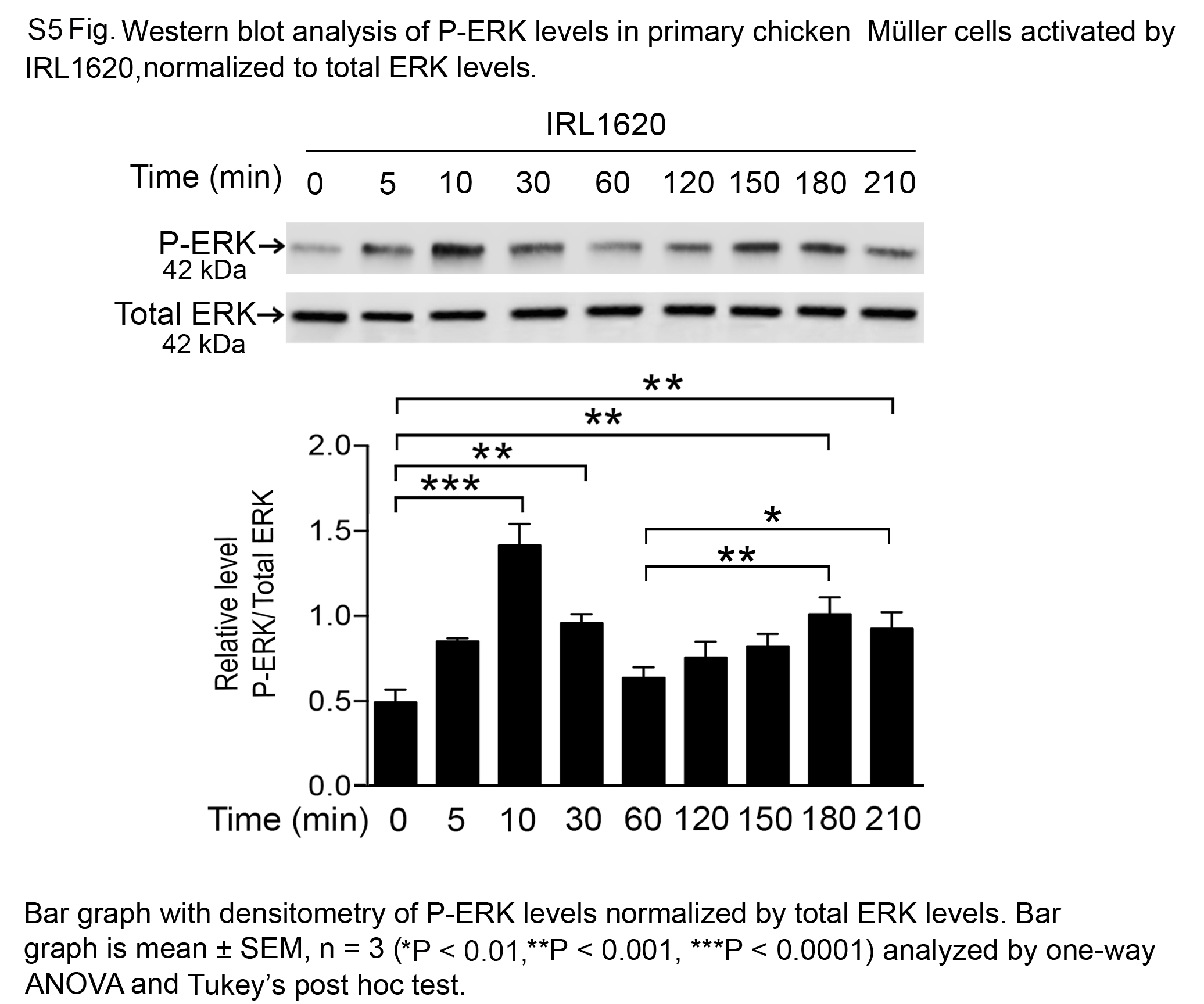

Supplement: S5 Fig — (TIF) [file pone.0167778.s005.tif]

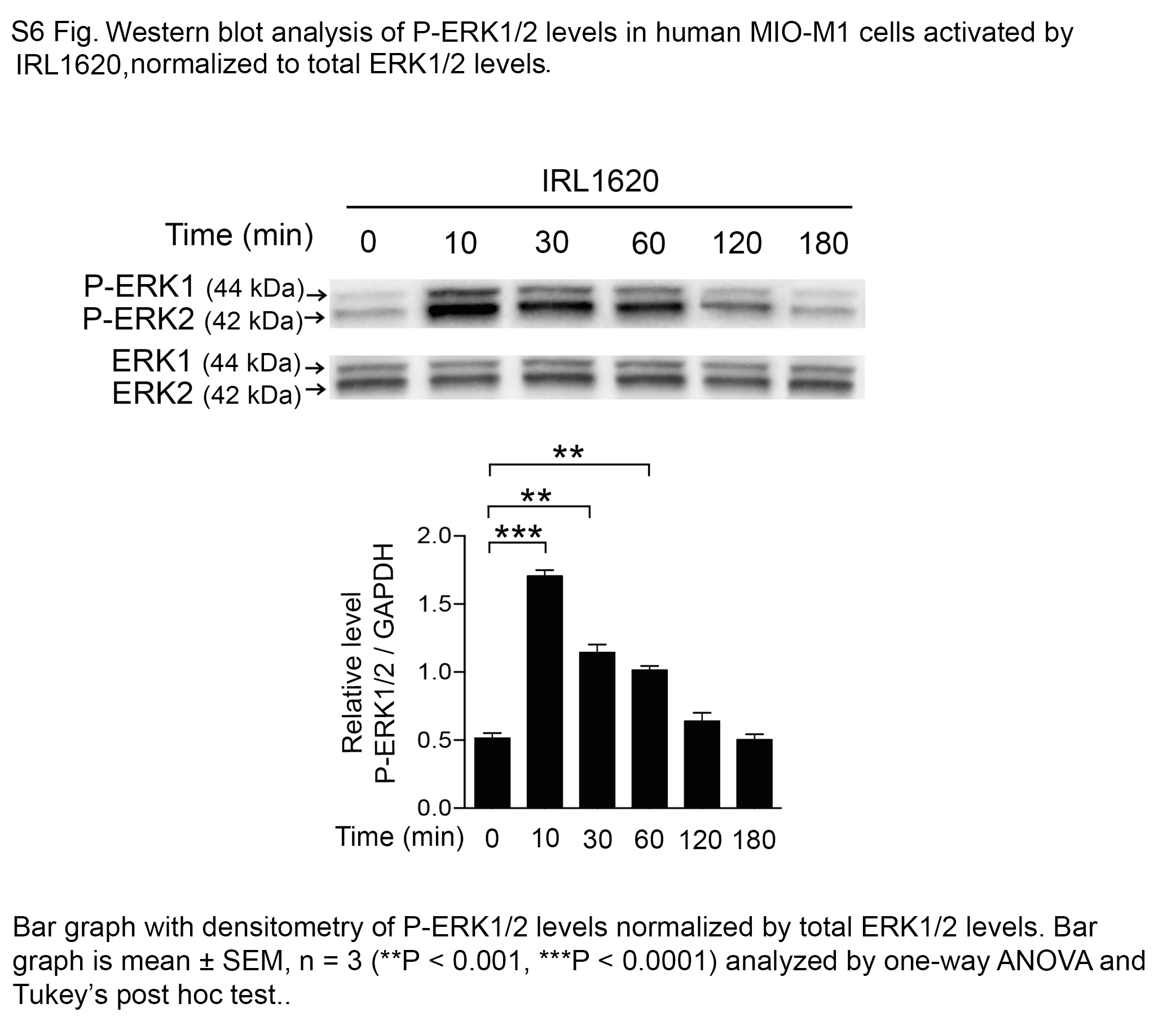

Supplement: S6 Fig — (TIF) [file pone.0167778.s006.tif]
