# Supplementary material for: Endothelin B Receptors on Primary Chicken Müller Cells and the Human MIO-M1 Müller Cell Line Activate ERK Signaling via Transactivation of Epidermal Growth Factor Receptors
Source: PLoS One. 2016 Dec 8;11(12):e0167778. doi: 10.1371/journal.pone.0167778 (PMC5145189; doi:10.1371/journal.pone.0167778)
Supplement: S1 Table — (PDF) [file pone.0167778.s007.pdf]

S1 Table. List of reagents and inhibitors

**Reagent**	**Catalog and company**	**Target**	**Action**
IRL1620	# 1196, Tocris Bioscience	Endothelin B receptor	Highly selective Endothelin B receptor agonist (Takai et al., 1992).
BQ-788	#1500, Tocris Bioscience	Endothelin B receptor	Potent, selective Endothelin B receptor antagonist (Ishikawa et al., 1994).
Recombinant Human EGF	# AF-100-15, Peprotech	EGF Receptor	EGF Receptor ligand. (Carpenter and Cohen, 1990).
PP1	#P0040-5MG, Sigma Aldrich	Cytosolic Src kinase	A potent and highly selective Src kinase inhibitor (Hanke et al., 1996).
PP2	#1407, Tocris Bioscience	Cytosolic Src kinase	A potent and highly selective Src kinase inhibitor (Hanke et al., 1996).
GM6001	#BML-EI300-0001, *ENZO* Life Science	Matrix metalloproteinases (MMPs)	A potent broad-spectrum MMPs inhibitor (Santiskulvong and Rozengurt, 2003).
AG1478	#1276, Tocris Bioscience	EGF Receptor-kinase	A highly potent EGF receptor-kinase inhibitor (Han et al., 1996).

References:

Carpenter, G., Cohen, S., 1990. Epidermal growth factor. The Journal of biological chemistry 265, 7709-7712.

Han, Y., Caday, C.G., Nanda, A., Cavenee, W.K., Huang, H.J., 1996. Tyrphostin AG 1478 preferentially inhibits human glioma cells expressing truncated rather than wild-type epidermal growth factor receptors. Cancer Res 56, 3859-3861.

Hanke, J.H., Gardner, J.P., Dow, R.L., Changelian, P.S., Brissette, W.H., Weringer, E.J., Pollok, B.A., Connelly, P.A., 1996. Discovery of a novel, potent, and Src family-selective tyrosine kinase inhibitor. Study of Lck- and FynT-dependent T cell activation. The Journal of biological chemistry 271, 695-701.

Ishikawa, K., Ihara, M., Noguchi, K., Mase, T., Mino, N., Saeki, T., Fukuroda, T., Fukami, T., Ozaki, S., Nagase, T., et al., 1994. Biochemical and pharmacological profile of a potent and selective endothelin B-receptor antagonist, BQ-788. Proceedings of the National Academy of Sciences of the United States of America 91, 4892-4896.

Santiskulvong, C., Rozengurt, E., 2003. Galardin (GM 6001), a broad-spectrum matrix metalloproteinase inhibitor, blocks bombesin- and LPA-induced EGF receptor transactivation and DNA synthesis in rat-1 cells. Exp Cell Res 290, 437-446.

Takai, M., Umemura, I., Yamasaki, K., Watakabe, T., Fujitani, Y., Oda, K., Urade, Y., Inui, T., Yamamura, T., Okada, T., 1992. A potent and specific agonist, Suc-[Glu9,Ala11,15]-endothelin-1(8-21), IRL 1620, for the ETB receptor. Biochemical and biophysical research communications 184, 953-959.
