# Supplementary material for: Endothelin B Receptors on Primary Chicken Müller Cells and the Human MIO-M1 Müller Cell Line Activate ERK Signaling via Transactivation of Epidermal Growth Factor Receptors
Source: PLoS One. 2016 Dec 8;11(12):e0167778. doi: 10.1371/journal.pone.0167778 (PMC5145189; doi:10.1371/journal.pone.0167778)
Supplement: S2 Table — (PDF) [file pone.0167778.s008.pdf]

S2 Table. List of primary and secondary antibodies.

Antibody name	Antigen, dilution and purpose	Catalog and Company	Host
GAPDH	Glyceraldehyde 3-phosphate dehydrogenase, 1:15000 for WB	# ab9482, HRP conjugated, Abcam	Mouse
P-ERK	Phospho-ERK1/2 MAPK, 1:200 for IHC/ICC, 1:000 for WB.	# 9101, Cell Signaling Technologies	Rabbit
Total ERK	ERK1/2 MAPK, 1:1000 for WB.	# 4695, Cell Signaling Technologies	Rabbit
2M6	2M6 antigen (TopAP), Müller glial marker, 1:200 for IHC/ICC.	Dr Paul Linser, University of Florida (Ochrietor et al., 2010; Schlosshauer et al., 1991)	Mouse
pEGFR (Y-1173)	Phospho-EGF receptor (Try 1173), 1:1000 for WB.	# sc-12351-R, Santa Cruz Biotechnology	Rabbit
EGFR	EGF receptor, 1:1000 for WB	# sc-03, Santa Cruz	Rabbit
Mouse 2nd antibody	Mouse IgG, 1:1000 for IHC/ICC	# A10037, Alexa 568, Invitrogen	Donkey
Rabbit 2nd antibody	Rabbit IgG, 1:1000 for IHC/ICC	# A21206, Alexa 488, Invitrogen	Donkey
Rabbit 2nd antibody	Rabbit IgG, 1:25,000 for WB	# ab97064, HRP conjugated, Abcam	Donkey

Note: IHC, immunohistochemistry; ICC, immunocytochemistry; and WB, western blotting.

Reference:

Ochrietor, J.D., Moroz, T.P., Linser, P.J., 2010. The 2M6 antigen is a Muller cell-specific intracellular membrane-associated protein of the sarcolemmal-membrane-associated protein family and is also TopAP. Molecular vision 16, 961-969.

Schlosshauer, B., Grauer, D., Dutting, D., Vanselow, J., 1991. Expression of a novel Muller glia specific antigen during development and after optic nerve lesion. Development 111, 789-799.
