# Supplementary material for: Endothelin B Receptors on Primary Chicken Müller Cells and the Human MIO-M1 Müller Cell Line Activate ERK Signaling via Transactivation of Epidermal Growth Factor Receptors
Source: PLoS One. 2016 Dec 8;11(12):e0167778. doi: 10.1371/journal.pone.0167778 (PMC5145189; doi:10.1371/journal.pone.0167778)
Supplement: S3 Table — (PDF) [file pone.0167778.s009.pdf]

S3 Table. List of QRT-PCR primers sequences

Primer name	Primer sequences (5´-3´)<br>F- forward, R- reverse	Ensembl Transcript ID
Chicken<br>β-ACTIN	F- AGGTCATCACCATTGGCAATG<br>R- CCCAAGAAAGATGGCTGGAA	ENSGALT00000015673
Chicken<br>EDNRA	F- CCATTGGTTTTGCCGTGGTA<br>R- CCACCAATCCTTTGCATCTTTG	ENSGALT00000016263
Chicken<br>EDNRB	F- GCTGAAGACCTAAGCACATGCA<br>R- TGCAAGAAAGGTGGTAACAGCA	ENSGALT00000036512
Chicken<br>EDNRB2	F- CTCCCCTTAGTATGCACTGGCA<br>R- CGCCGTTTCATGTGGTCATT	ENSGALT00000012072
Chicken<br>EDN1	F- TGCAGCAGGACAAAACGCT<br>R- TGGATTTCCCTTCTCCCCAT	ENSGALT00000020785
Chicken<br>EDN2	F- GGAGCAGGTGCAAACCAAGAT<br>R- TCCACTCATGGCCTTTCCTTC	ENSGALT00000000942
Chicken<br>EDN3	F- GAAGCTCTCTCCATGCTCCCTT<br>R- CTGTTGCTGAAATTTGGATCCG	ENSGALT00000043015
Chicken<br>HB-EGF	F- AAGGCCCAGTTACTCCCAA<br>R- AATCCTTGTACTTCCGCAG	ENSGALT00000001401
Chicken<br>SOX2	F- GTCACCTCCTCGTCTCATTCG<br>R- GGCAGCTGGTTCTGGTACTTC	ENSGALT00000014379
Human<br>β-ACTIN	F- ATGGCCACGGCTGCTTCCAGC<br>R- CATGGTGGTGCCGCCAGACAG	ENST00000331789
Human<br>EDNRA	F- AGGACAGCATGAACTGACCACC<br>R- GGATCAGAGAAGAGATTCCCGG	ENST00000324300
Human<br>EDNRB	F- TGGCCATTTGGAGCTGAGA<br>R- TCCAAGAAGCAACAGCTCGAT	ENST00000377211
Human<br>EDN1	F- CAGCGTCCTCGTTCAAAACATT<br>R- CCCCAGATGAAAGAAGAGACCA	ENST00000379375
Human<br>EDN2	F- CACAGTCAAGAGCCTCTTTGCC<br>R- CAATGTTCCTCCAGCTCACGA	ENST00000372587
Human<br>EDN3	F- CCAAACTCTGGACGTCAGCAGT<br>R- TGAGCTTTGGATGGTGGAGGT	ENST00000311585
Human<br>HB-EGF	F- CCGTGGTGGCTGTGGTGCTGT<br>R- GCAGTCCCCAGCCGATTCCTT	ENST00000230990
Human<br>CRALBP	F- TCCGGACTTCAGATCTCAGGAA<br>R- AGGTCGTGGTGAAGTACCATGG	ENST00000268125
